# Statistical heartburn: an attempt to digest four pizza publications from the Cornell Food and Brand Lab

**DOI:** 10.1186/s40795-017-0167-x

**Published:** 2017-07-10

**Authors:** Tim van der Zee, Jordan Anaya, Nicholas J. L. Brown

**Affiliations:** 1 Graduate School of Teaching (ICLON), Leiden, Netherlands; 2 Omnes Res, Charlottesville, USA; 3 University Medical Center, University of Groningen, Groningen, Netherlands

**Keywords:** Statistics, Reproducibility, Replication, Reanalysis

## Abstract

**Background:**

We present the results of a reanalysis of four articles from the Cornell Food and Brand Lab based on data collected from diners at an Italian restaurant buffet.

**Method:**

We calculated whether the means, standard deviations, and test statistics were compatible with the sample size. Test statistics and *p* values were recalculated. We also applied deductive logic to see whether the claims made in each article were compatible with the claims made in the others. We have so far been unable to obtain the data from the authors of the four articles.

**Results:**

A thorough reading of the articles and careful reanalysis of the results revealed a wide range of problems. The sample sizes for the number of diners in each condition are incongruous both within and between the four articles. In some cases, the degrees of freedom of between-participant test statistics are larger than the sample size, which is impossible. Many of the computed *F* and *t* statistics are inconsistent with the reported means and standard deviations. In some cases, the number of possible inconsistencies for a single statistic was such that we were unable to determine which of the components of that statistic were incorrect. Our Appendix reports approximately 150 inconsistencies in these four articles, which we were able to identify from the reported statistics alone.

**Conclusions:**

We hope that our analysis will encourage readers, using and extending the simple methods that we describe, to undertake their own efforts to verify published results, and that such initiatives will improve the accuracy and reproducibility of the scientific literature. We also anticipate that the editors of the journals that published these four articles may wish to consider whether any corrective action is required.

## Background

Concerns have been raised about the reproducibility of scientific research [1], with many recent concerns focusing on psychological research [2]. Commonly cited reasons for the reproducibility crisis include the use of small sample sizes leading to low statistical power [3], a culture of questionable research practices [4], and an incentive structure that rewards large numbers of publications reporting sensational findings with little penalty for being wrong [5, 6]. However, a number of recent articles lead us to question whether a certain fraction of non-reproducibility might be due to simple reporting or calculation errors. The prevalence of errors in the *p* values associated with reported test statistics has been estimated to be anywhere from around 18% to almost 50% [7, 8]. Another study found at least one apparent error in something as simple as a reported mean in around 50% of the articles its authors examined [9]. Of course, it is possible for one or two elementary typos to occur during the drafting of an article, and an erroneous statistic due to a participant missing a response on an item can slip into even the most carefully proofread manuscript. However, the presence of a high number of errors in simple statistics might make the reader wonder what else might have been done in a less than rigorous fashion.

Here, we examine four articles from the same laboratory, which contain a remarkably high number of apparent errors and inconsistencies. Our attention was first drawn to this series of articles when the senior author wrote a blog post about the context in which the articles came to be written [10]. When we followed the references to the articles cited in that blog post we immediately noticed some apparent inconsistencies^1^. We therefore decided to perform a detailed reanalysis of the four articles that seemed to be closely related to each other, to see whether any other problems might emerge. A detailed list of approximately 150 individual inconsistencies and other problems is given in the Appendix; within the text of this article we discuss some of the overarching issues with the four target articles and the implications of what we found.

### The articles in question

We reanalyzed four articles by Özge Sigirci, Brian Wansink, and their colleagues, which appear to be based on a single data set from one field experiment [11–14]. We will refer to these articles with the following numbering:

1. Just, D. R., Sigirci, Ö., & Wansink, B. (2014). Lower buffet prices lead to less taste satisfaction. *Journal of Sensory Studies*, *29*(5), 362–370. doi:10.1111/joss.12117
2. Just, D. R., Sigirci, Ö., & Wansink, B. (2015). Peak-end pizza: Prices delay evaluations of quality. *Journal of Product & Brand Management*, *24*(7), 770–778. doi:10.1108/jpbm01-2015-0802
3. Kniffin, K. M., Sigirci, Ö., & Wansink, B. (2016). Eating heavily: Men eat more in the company of women. *Evolutionary Psychological Science*, *2*(1), 38–46. doi:10.1007/s40806-015-0035-3
4. Sigirci, Ö., & Wansink, B. (2015). Low prices and high regret: How pricing influences regret at all-you-can-eat buffets. *BMC Nutrition*, *1*(1), 36. doi:10.1186/s40795-015-0030-x

Each of the four target articles describes what we believe to be the same field study. Apart from the blog post mentioned above, which strongly implied that the data set is common to these four articles, we base this conclusion on the following observations:

- Articles 1, 2, and 4 state that the study took place at “Aiello’s Italian Restaurant, a restaurant mid-way between Syracuse and Binghamton, New York.” Article 3 describes the location, differently but not inconsistently, as “an Italian restaurant in Northeastern USA.”
- All four articles mention that the study took place over a two-week period. Articles 1, 2, and 4 further specify that this period was in the spring, with data being collected between 11:00 a.m. and 1:30 p.m., and with the weather being overcast and chilly or rainy throughout the days of the study.
- All four articles describe the presence of an all-you-can-eat lunch buffet. Articles 1, 2, and 4 (but not 3) explain that the study used a randomized between-subjects design in which participants were given a flyer that entitled them to pay either $4 or $8 for the lunch buffet and a free beverage. People who arrived in groups were all assigned to the same coupon condition.
- Articles 1, 2, and 4 describe the buffet as consisting of pizza, salad, breadsticks, pasta, and soup. Article 3 mentions “pizza, salad, and side dishes.”
- All four articles mention that the number of people recruited was either 139 in total, or 133 adults. Article 1 reports that of 139 total participants, 6 were eliminated for being under 18 years of age, thus also giving a total of 133 adults.
- All four articles describe how participants were intercepted at the cash register and given a short questionnaire, which asked for demographic information along with a variety of questions about their restaurant experience.

Given the identical setting, which is often described with identical sentences across the four articles, and the presence of many identical results in different articles (e.g., Table 1 has been copied verbatim between Articles 1 and 2), we conclude that these articles all describe the same field study. However, none of the articles mentions that they are based on the same data set as their predecessors, even though they were published over a period of many months. We consider that this may constitute a breach of good publication ethics practice [15]; it is important for the reader to know that other articles may exist based on the same data set, so that he or she may appropriately judge the independent claims of each article.

**Table 1:** Relevant data from Article 4, Table 2

## Method

None of the four target articles provides a link to a public version of the data set. We wrote to the corresponding authors of all four articles, asking explicitly for a copy of the data. We received only one reply, from the authors’ lab’s “Communications Specialist”; this reply did not address our request for the data and instead suggested that we conduct a replication of the study. We wrote back, emphasizing that we wished to check a number of apparent inconsistencies in the articles, but after two weeks we still have not heard back^2^. We note that the publisher of at least one of the four target articles, BioMed Central (Article 4 was published in *BMC Nutrition*), currently imposes an explicit requirement on authors to share their data as a condition of publication: “Submission of a manuscript to a BioMed Central journal implies that materials described in the manuscript, including all relevant raw data, will be freely available to any scientist wishing to use them for non-commercial purposes” [16]. Fortunately, multiple techniques exist that can identify a number of errors in reported statistics even without access to the original data.

### Granularity errors

Statistics of discrete data are *granular*, with the effect that they can only take on certain values when rounded to any given number of decimal places (typically 2). When the granularity of the statistic is greater than the precision of the reported values, it becomes possible to report values that are mathematically impossible. For example, if the data are reported as integers, such as survey questions on a Likert-type scale, and the mean is reported to two decimal places (a precision of 0.01), it is possible for reported means to be inconsistent if the sample size is below 100 [9]. Similarly, it is also possible for standard deviations (SDs) to be inconsistent, although the calculation of which values are consistent or not is more complex [17].

Almost all of the means and SDs in the four target articles were for (sub)sample sizes below 100 and reported to two decimal places, allowing us to scrutinize them with granularity testing. To do this, we used a web application (available at http://www.prepubmed.org/grimmer_sd) that simultaneously checks means (GRIM) and standard deviations (GRIMMER), as explained by [17]; we also checked means independently using an Excel spreadsheet (available at https://osf.io/3fcbr). SDs were assumed to be sample (versus population) SDs in all cases. We took a conservative approach to rounding, allowing potentially ambiguous values (e.g., a mean of 0.125) to be rounded both up and down [9].

### *P* values

We performed an automated check of the consistency between test statistics and *p* values with *statcheck* [18], which did not identify any errors in any of the four articles. However, it should be noted that *statcheck* was unable to identify most of the test statistics (it does not search tables and can miss tests in the text that are not in APA format). Furthermore, *statcheck* only checks if the *p* value is consistent with the test statistic and degrees of freedom (DFs); it cannot check if the test statistic or DFs are themselves correct.

### Test statistics

It is possible to recalculate the test statistics from a *t* test or an ANOVA using only the per-cell sample sizes, means, and standard deviations. The rpsychi package in R [19] provides functions to do this for one- and two-way ANOVAs (a one-way ANOVA with two groups is equivalent to a *t* test, with the *F* statistic being the square of the *t* statistic).

To account for uncertainty in the recalculation of the test statistics due to errors introduced by the rounding of the reported means and SDs on which these statistics were based, we calculated upper and lower bounds for the test statistics, and treated reported statistics in the target articles as valid if they fell within these bounds. We calculated the upper bound of each *F* (or, in a few cases, *t*) statistic by subtracting.005 from all of the SDs (thus biasing all of the standard errors downward) and then generating every combination of the means with.005 either added to or subtracted from each, retaining the largest test statistic produced by all of these combinations. For the lower bound, we performed the analogous operations in reverse, adding.005 to each of the SDs and retaining the smallest test statistic from every set of means that had been adjusted by either the addition or subtraction of.005 to each value. Because we used all of the most extreme possible values from which the means and SDs could have been rounded, we believe that our reanalysis gives a conservative estimate of the number of inconsistencies in the *F* and *t* statistics reported in the four target articles.

As well as the bounds for the *F* and *t* statistics, we also recalculated *p* values wherever this was possible and appropriate. All of the recalculations were performed separately by the second and third authors in Python and in R, respectively, and checked by all authors. We copied and pasted the means and SDs from the published tables into a text editor and then transformed these numbers to data structures in the programming languages that we were using, in order to avoid possible corruption due to typing errors on our part. The code for our calculations is available in the GitHub repository (https://github.com/OmnesRes/pizzapizza) associated with the present article.

## Results

Here we will go over some observations we made which both raise serious questions about the integrity of the data set used in these papers, and the accuracy of the report.

### Inconsistent sample sizes within and between articles

Across the four articles we identified a number of cases where the reported sample sizes were inconsistent, either with the associated means and SDs, or simply with each other across tables. Here we present an example where the accumulation of multiple inconsistencies across articles makes it difficult to identify which of the reported statistics are correct and which are erroneous.

Below we reproduce Table 2 from Article 4. This table lists the means and (in parentheses) SDs of responses to a series of Likert-type items scale. Numbers in red denote means and SDs that are inconsistent with the stated sample size at the top of each column.

Although granularity testing typically involves the use of a calculator or spreadsheet program [9] for means, or more sophisticated software for SDs [17], some of these errors can be checked by simple visual inspection. For example, with a sample size of 10 any mean reported to two decimal places must always have a zero in the second decimal place; yet, this table contains means of 2.25 and 3.92 for a sample size of 10 ($8 condition, 3 pieces of pizza). Overall, it seems to us that the number of granularity errors in this table is indicative of problems that go beyond simple transcription errors.

Table 3 of Article 4 is, in principle, simply a modified version of Table 2 with a different nesting of conditions (buffet price nested within pieces of pizza consumed, versus pieces of pizza nested within buffet price in Table 2). The two tables should, therefore, have identical summary statistics for each sub-column. However, we found many discrepancies between the two tables, as shown in this reproduction.

**Table 2:** Relevant data from Article 4, Table 3

The values in red are those that are different between Tables 2 and 3. There is no obvious pattern to these differences. Of particular concern here are the inconsistent sample sizes. Inspecting the sample sizes in Table 2 reveals that they do not add up to 95 (18+18+7+17+19+10=89), which is the stated number of total diners in this study. In Table 3 the sample sizes do add up to 95 (18+19+18+21+7+12=95).

**Table 3:** Relevant data from Article 2, Table 2

Half price ($4) models					Full price ($8) models					
Beginning	Total	End	Peak	Peak-end		Beginning	Total	End	Peak	Peak-end
*N* = 62	*N* = 41	*N* = 47	*N* = 62	*N* = 47		*N* = 60	*N* = 26	*N* = 38	*N* = 60	*N* = 38

We attempted to resolve some of our questions about the sample sizes by examining the DFs in the text of the article (p. 3). The *F* statistics mentioned in the text appear to correspond to a series of one-way ANOVAs that are not summarized in the tables. The apparent 3 by 1 ANOVAs discussed have the DFs (2,84). This implies a total sample size of 84+3=87, which is different from both the total of 89 calculated above from the column *N*s in Table 2 and the total of 95 that is mentioned in the text and reported in Tables 1 and 3. The text also mentions an apparent 2 × 1 ANOVA with DFs (1,84), implying a total sample size of 84+2=86.

A certain amount of variation in sample sizes and (especially) DFs can be expected in studies where people fail to respond to some items, since statistical software will typically omit (via listwise deletion) participants who are missing a value on one variable. However, we are unable to imagine that such occasional dropping of one or two participants could account for all of the discrepancies that we identified in Article 4. There is nothing in the text of that article to suggest a reason for the reported sample sizes of the sub-columns to be different between Tables 2 and 3, and yet half of these per-column *N*s are inconsistent across the two tables.

We checked the stated overall sample sizes in all four publications. An immediately observable inconsistency is that Articles 1 and 2 include 122 diners, while Article 3 includes 105 diners, and Article 4 includes 95 diners. These differences were only partially explained by the authors. Taking into account information from all four articles, it appears that a total of 139 participants were initially recruited, but six were under 18 years of age and excluded from all of the studies, leaving 133 eligible participants. Article 1 reported that a further 11 people did not complete all of the survey questions, giving a total of 122 participants for whom complete information was available; this is presumably also the source of the sample size of 122 in Article 2. However, Article 3, in which the focus was the effect of the sex of participants’ table companions on their eating patterns, states that 20 people did not complete the survey, and a further eight diners were excluded because they ate alone, giving a final sample size of 105. The reason for the discrepancy in the number of people who did not complete the survey (11 versus 20) is not clear.

In Article 4, there is no mention of any incomplete questionnaires. The explanation given for how the sample size of 95 was arrived at, from the initial recruitment of 139 people, is that this was the number of “respondents who ate at least one piece of pizza and were included in the analysis” (p. 2). At first sight, this phrase could be taken to suggest that a substantial number of participants — perhaps, based on Articles 1 and 2, (122−95)=27 — did not eat any pizza. However, from Table 2 of Article 1 and Table 2^3^ of Article 2, it is clear that all 122 diners who completed the survey ate at least 1 piece of pizza. This leads us to believe that the criterion for being “included in the analysis” of Article 4 was not eating at least 1 piece of pizza, but rather, eating *no more than 3* pieces of pizza. This appears to be confirmed by the sum of the number of diners in each condition in Table 3 of Article 4. However, this criterion seems strange since the stated purpose of the study in Article 4 was to examine the extent to which “how much consumers pay for their food influences their perceptions about satiety, feelings of guilt and overeating” (p. 3). It seems rather illogical to exclude from such a study precisely those people who ate the *largest* amount of pizza. (We also wonder, in passing, why the authors assigned a special status to pizza, while apparently ignoring the effect that consumption of another high-calorie food such as pasta might have on diners’ feelings about overeating. That is, those who ate no pizza at all, or just one slice, might have eaten much more food of other types, which seems relevant for a study that is at least notionally about overeating in general).

We continued our analyses of the sample sizes to determine how many people in each price condition (i.e., paying either $4 or $8 for the buffet) ate each number of slices^4^ of pizza. We started this process in Table 2 of Article 2, which provides statistics for different linear regression models predicting variance in diners’ overall satisfaction with the pizza that they ate from their satisfaction with each slice.

Here, the “Beginning” model is built with information regarding the first slice of pizza that each diner ate. The “End” model is built with information regarding the last slice that each diner ate, provided they ate at least 2 slices. The “Total” model contains, for diners who ate at least 3 slices, ratings of the first slice, middle slice (or one of the pair of slices on either side of the “midpoint”, if the total number of slices was even), and last slice. That is:

- The “Beginning” model contains all diners who ate at least 1 slice
- The “End” model contains all diners who ate at least 2 slices
- The “Total” model contains all diners who ate at least 3 slices

With this information it follows that at the $4 price point, 62 diners ate 1 or more slices, 47 diners ate 2 or more slices, and 41 diners ate 3 or more slices. From this we can deduce that (62−47)=15 diners ate exactly 1 slice and (47−41)=6 diners ate exactly 2 slices. Similarly, at the $8 price point, 60 diners ate 1 or more slices, 38 diners ate 2 or more slices, and 26 diners ate 3 or more slices, meaning that (60−38)=22 diners ate exactly 1 slice and (38−26)=12 diners ate exactly 2 slices.

We were able to compare these per-condition sample sizes for people who ate exactly 1 or 2 slices with the equivalent numbers from Table 3 of Article 4. (We chose Table 3, rather than Table 2, because the per-column sample sizes in Table 3 are consistent with the overall sample size of 95 for that study). Our Table 4 shows the sample sizes, all of which are different between the two articles. Additionally, the sample in Article 4 appears to be a subset of the sample from Article 2 (with people who ate more than 3 slices of pizza excluded); as a result, it should not be possible for any of the groups in Article 4 to be larger than the equivalent group in Article 2. However, three out of the four groups are larger in Article 4 than in Article 2. Even if the diners in Article 4 are not a subset of those in Article 2 (for example, because the 11 diners who were excluded from Article 2 for having incomplete questionnaires were included in Article 4, and all 11 of those diners ate exactly 2 slices of pizza), this is insufficient to explain the number of people reported as eating exactly 2 slices of pizza in Article 4.

**Table 4:** Comparison of sample sizes for 1 and 2 pieces of pizza between Articles 2 and 4

	1 Piece			2 Pieces	
	$4	$8		$4	$8
Article 2	15	22		6	12
Article 4	18	19		18	21

A further discrepancy in the reported sample sizes across these articles is apparent when we examine Article 1, although Table 3 of this article provides neither sample sizes nor SDs to go with the reported means and *F* statistics. The degrees of freedom on the *F* statistics reported in the text on pp. 365–366, such as (1,35), imply that the number of people who ate at least 3 pieces of pizza was no greater than 37. Some of these *F* tests have even lower DFs; we assume that this may be because a small number of participants (three for “satisfaction” and two for “enjoyment”) did not provide a rating on this dimension for some or all of the slices of pizza that they ate, although the article makes no mention of any missing data. There is thus a considerable difference between this *N* and the sample size of 67 (41 in the $4 condition and 26 in the $8 condition) reported for exactly the same criteria — that is, people who ate at least three slices of pizza — in Table 2 of Article 2.

One possible explanation for this could be if in Article 1, instead of diners who ate *at least* 3 pieces of pizza, the reported ratings are from diners who ate *exactly* 3 pieces of pizza, so that the “first/middle/last” ratings could be mapped more easily onto “1st/2nd/3rd” (see also our next section, entitled “First, middle, last, or 1st, 2nd, 3rd?”). However, even here the number of diners is difficult to reconcile with the other articles. For example, Article 4 suggests that only 17 (Table 2) or 19 (Table 3) people ate exactly 3 slices.

### First, middle, last, or 1st, 2nd, 3rd?

Table 2 in Article 1 describes diners’ ratings for the “first”, “middle”, and “last” slice of pizza. However, the definition of what constitutes the middle or last slice is not provided in Article 1. An examination of Article 2 reveals the definition of a middle slice, and also that someone who only ate 1 slice has a first slice rating, but not a last slice rating (i.e., the “only” slice is, somewhat arbitrarily, designated as the first, although it could arguably also be described as the last slice with equal validity). Despite the fact that the questionnaire asked people to describe their ratings of the first, middle, and last slices (Article 2, p. 772), Table 3 of Article 1 compares diners’ evaluations of the 1st piece, 2nd piece, and 3rd piece, as does Figure 1 of the same Article. Article 2 exclusively uses the terms “first”, “middle”, and “last”, and only defines a middle slice for diners who ate 3 or more slices. A diner who ate an odd number of slices has a clear middle slice, but diners who ate an even number of slices (above 2) could choose one of two possible candidates as the middle slice.

Given these definitions, it is not clear to us how some diners could have had a rating for a “2nd” slice. Articles 1 and 2 both make it clear that diners were asked about their evaluations of the first, middle, and last slices. For example, if a diner ate 5 slices, they would have a first slice rating which would be slice 1, a middle slice rating which would be slice 3, and a last slice rating which would be slice 5. And as the authors’ definition states, someone who ate 4 slices could have chosen either slice 2 or slice 3 as their middle slice. But Article 2 does not state whether what we might call the “absolute slice sequence number” (e.g., for someone who ate 4 slices, this would be either 2 or 3) of the middle slice was recorded.

Despite the fact that some diners who ate 4 or more slices may not even have given a rating for their 2nd slice, Table 3 of Article 1 reports evaluations of the 1st, 2nd, and 3rd slice for “diners who ate at least three pieces” (p. 365). Perhaps this use of ordinal numbers is an error due to copying and pasting during the preparation of multiple manuscripts, since Article 4 discusses diners who ate 1, 2, or 3 slices of pizza; it could be that in Table 3 of Article 1, and the discussion around it, the terms “1st,” “2nd,” and “3rd” should have read “first,” “middle,” and “last.” But if this were the case, then Figure 1A of Article 1 ought to show identical results to Figure 1 of Article 2; however, these figures differ in several ways (for example, the rating for the taste of the last slice in the $4 condition is 6.10 in Figure 1 of Article 2, but the bar in Figure 1A of Article 1, and the corresponding entry in Table 3 of Article 1, shows a value of 6.38). As a result, it is unclear to us exactly what data are presented in Table 3 and Figure 1 of Article 1.

Further investigation into Articles 1 and 2 only adds to the confusion. On page 772 of Article 2 the authors use the DFs (2,80) and (2,50) to test the effect of slice order (first, middle, last) on pizza evaluations for the half price and full price groups, respectively. These DFs are correct assuming a repeated measures design and taking the sample sizes from the “total” models (41 and 26) in Table 2 of Article 2. In turn, this implies that Figure 1 of Article 2 shows the evaluations of diners who ate 3 or more slices of pizza. While someone who ate a first slice could have consumed 1, 2, 3, or more slices, anyone who ate a middle slice must have eaten 3 or more slices (by definition); as a result we should be able to obtain the “taste of middle slice” value for Figure 1 of Article 2 from Table 2 of Article 1. The value for the half-price ($4) group from Table 2 of Article 1 is 6.68, which matches 6.68 in Figure 1 of Article 2. However, the value for the full-price ($8) group of 7.97 in Table 2 of Article 1 does not match the value of 8.00 in Figure 1 of Article 2.

### Further inconsistencies across the four articles

Assuming that all four articles do indeed describe studies using the same participants and the same data collection procedures, there appear to be further inconsistencies in the reporting of the methods and results across the four articles. For example:

1. Article 3 describes how researchers observed the diners during their meal in order to establish how many slices of pizza and bowls of salad each person ate, including “appropriate subtractions” (p. 41) for unfinished slices of pizza and bowls of salad when these were cleaned away by waitstaff. However, none of the other articles gives any indication that the number of slices of pizza consumed was anything but an integer. Additionally, Article 2 states that the researchers were “not able to accurately measure consumption of non-pizza food items” (p. 772) — a statement that might refer to an inability to count fractional portions, or simply explain the article’s exclusive focus on pizza, but which in any case directly contradicts Article 3, which included data for how much salad was consumed and claimed that adjustments were made for unfinished bowls.
2. Article 2 claims that “pizza was by far the most popular choice on the buffet”; however, Table 2 in Article 3 indicates that more bowls of salad per person than slices of pizza were consumed by every group, and sometimes by a wide margin (e.g., 4.83 bowls of salad vs. 1.33 pizza slices for females eating with males). Hence, we are not entirely sure how this claim about the relative popularity of pizza can be justified.
3. Articles 1, 2, and 4 all state that the modal number of slices of pizza consumed by each participant was three. However, the numbers of participants who were reported to have consumed each number of slices varies considerably between these articles. In Article 1, the DFs on pp. 365–366 suggest that 37 people (see previous discussion “First, middle, last, or 1st, 2nd, 3rd?”) ate three or more slices, and the per-column *N*s of Table 2 show that 122 people ate at least 1 slice, meaning that (122−37=85) people ate either 1 or 2 slices. However this number might be partitioned between those who ate 1 and 2 slices, at least one of the components will be 43 or larger (i.e., greater than the 37 people who ate 3 slices), so the modal number of slices must be either 1 or 2. In Article 4, the claim that the modal number of slices was 3 is directly contradicted by the sub-column headings in Tables 2 and 3, regardless of how the inconsistencies between these two tables are resolved (see also the “Inconsistent sample sizes within and between articles” section above). Only in Article 2 is it possible for the modal number of slices of pizza consumed to have been 3, given the reported sample sizes. Thus, either the modal number of slices consumed was different between studies and has been reported incorrectly in at least one article (which would be an interesting result, given all the evidence that these four articles almost certainly come from the same data set), or the numbers of participants who ate each number of slices of pizza are incorrectly reported by a wide margin in at least two articles.

## Discussion

Here, we have presented in-depth reanalyses of four published articles from the same laboratory that reported a variety of analyses of what appeared to be the same data set, gathered in the field setting of an all-you-can-eat buffet restaurant. We have shown that these articles contain a very large number of apparent errors and inconsistencies. The types of errors include: impossible sample sizes within and between articles, incorrectly calculated and/or reported test statistics and degrees of freedom, and a large number of impossible means and standard deviations. In total, we identified approximately 150 inconsistencies and impossibilities in these four papers. Taken together, these problems make it difficult to have confidence in the authors’ conclusions.

In examining these articles we were conservative with our methods and tried to give the authors the benefit of the doubt at all stages. We made generous allowances for rounding, considered all possibilities for test statistics, and made multiple passes within and across the articles to try and identify the correct sample sizes. We checked granularity errors with an online web application, with an Excel sheet, with R code, and by hand. Two of us reconstructed the test statistics with two different programming languages and multiple statistical packages and formulas, and in some cases with online applications as well. We consulted several sources to ensure that we understood the correct degrees of freedom for all the tests that appeared to have been performed by the original authors (most statistics are not accompanied by any information as to what was being measured). We constructed test data sets and checked our methods with these.

None of us can remember encountering a set of articles with as many inconsistencies and unresolved questions in the basic reporting of results as in this case. Our best guess as to what might have happened is that the four articles started out as one single project and some wires became crossed when this project was being sliced up into publishable units. This might explain the strange mixture of “first,” “middle,” and “last” slices with “1st,” “2nd,” and “3rd” in Article 1, with both of those sequences only being defined in other articles in the series.

We noted earlier that our attention was drawn to this series of articles by a blog post written by the senior author [10]. Reactions by readers of that blog post mostly fell into one of two categories. Some were critical of the hiring and management policies of the laboratory that they felt were implied by the blog post, while others expressed skepticism about the ways in which the hypotheses tested by the researchers were generated. Although we have our own feelings about both of these issues, we have chosen to concentrate here only on the objective problems that we identified in the published literature, in the hope that the record will be corrected by whatever means the authors and the respective journal editors might consider appropriate. After all, as the aforementioned blog post stated, the resume of one of the authors will always have these papers on it.

## Conclusions

Science cannot be expected to always be “correct”, but it is expected to be done carefully and accurately. This is essential because science builds upon itself, and without a solid foundation future studies are doomed to fail; the cumulative advances in knowledge required for true progress are only possible when the scientific literature is trustworthy and accurate.

Using the discussed papers as an example, the present work contributes to this goal by:

1. Raising awareness that the published literature is not flawless, and that these flaws can include gross inconsistencies and impossibilities.
2. Showing that many such errors can be analyzed effectively without direct access to the original data with various tools.
3. Calling upon the scientific community — both editors and reviewers prior to publication of an article, and readers in general after publication — to more closely scrutinize reported results in the literature.

The first and third of these points have been investigated and discussed before in various publications [1, 2, 4, 7–9]. However, relatively little attention has been given to the second point, namely *how* critical readers can scrutinize reported results in the literature, and what tools are available for this purpose. In this paper we have demonstrated various such tools, which allow a critical appreciation of the veracity of reported results *even without direct access to the data*. These tools can be used in addition to the readers’ more qualitative judgment of a paper, such as regarding the appropriateness of sampling or choice of statistical tests. Much of the value of these tools lies in their objectivity, as they test the mathematical plausibility of reported results. We think that the existence and usage of these tools ought to be more widely known among authors, editors, reviewers, and anyone who “merely” reads the published literature, as also argued for by [20].

Although there is arguably no one-size-fits-all approach when it comes to checking papers, there are a variety of standard steps that can be used with many studies. Manual inspection can reveal inconsistencies in reported sample sizes within a paper, or between multiple papers based on the same data. These checks should also include inspection of reported degrees of freedom, which are straightforward to calculate for common analyses such as *t* tests and ANOVAs. Subsequently, *statcheck*, and various other online tools, allow the critical reader to easily check common statistics such as *z*, *t*, and *F* tests either one by one [21–23] or *en masse* [18]. In addition, any mean or SD based on granular data (such as Likert-type measures) can be checked for plausibility using GRIMMER [17]. A most relevant application is that to statistics describing Likert-type data, which are very common in the social sciences. Note that none of the tests mentioned above are limited to values reported in texts or tables; visualizations such as charts or path diagrams should also be carefully inspected.

It is important to consider that all these tests can do is assess the accuracy of *reported* results, which may or may not reflect the veracity of the underlying data or the appropriateness of the analyses that were performed. Even the most conscientious researcher will undoubtedly make mistakes from time to time. As such, we suggest that these tests be interpreted carefully and conservatively, and readers should take into account the number and types of any set of inconsistencies. On the other hand, even when reported results were generated by a completely random process, some of them will not be detected as being inconsistent by tools like *statcheck* and GRIM/GRIMMER. That is, these tests may not only give false positives in some cases (typos and other innocent errors), but also false negatives (failing to detect a true inconsistency). For example, if a mean of 4.28 with a sample size of 58 is misreported as 4.27, this will be detected by GRIM [9], but if the misreported value were 4.26, this would appear to be consistent. This added uncertainty should be considered when interpreting the range of inconsistencies, or lack thereof. All in all, these tools can be powerful aids to the critical examination of published results, but their output should always be interpreted with caution.

Finally, we want to emphasize that a critical inspection of the published literature should not be mischaracterized as a hobby for the overly cynical, nor as so-called “methodological terrorism”. On the contrary, carefully evaluating presented data is a cornerstone of scientific investigation, and it is only logical to apply this also to the published literature. If we are not willing to critically assess published studies, we also cannot guarantee their veracity.

## Endnotes

^1^ The blog post mentioned a total of five articles. Four of these were on the same topic and are the subject of the present article; the fifth was on an unrelated topic, and we do not consider it here.

^2^ While the present article was under review, the Cornell Food and Brand Lab made their dataset public via https://foodpsychology.cornell.edu/research-statement-april-2017. However, this dataset release and the accompanying reanalyses fail to address many of the inconsistencies described in the present article, and indeed introduce new inconsistencies between the original analyses, the released dataset, and the lab’s reanalyses. We have made our detailed comments on these reanalyses available at https://peerj.com/preprints/3025/.

^3^ This table is referred to as “Table II,” in Roman numerals, in Article 2 itself; we have changed this to Arabic numerals for simplicity.

^4^ Articles 1 and 4 refer principally to “pieces” of pizza; Articles 2 and 3 refer principally to “slices.” In the present article, we have attempted to use the word that most closely corresponds to the article being examined at any particular point.

## Appendix: List of inconsistencies

The following is a list of inconsistencies that we noticed and verified mathematically in the four publications. This list may not be exhaustive, as not all statistics could be checked without the data set and more details of the methods that were used. Sample sizes listed in the publications were taken at face value except for lines 5–10 of Article 1, Table 2, where it is clear from Article 2 that different numbers of diners have first slice, middle slice, and last slice data. We have no way of knowing whether the inconsistencies we identified are typos or calculation errors.

### Article 1: “Lower buffet prices lead to less taste satisfaction”

For Table 2 the sample sizes were determined from Article 2. For $4 buffet, rows 1–4: *N* = 62, rows: 5–7 *N* = 41, rows 8–10: *N* = 47. For $8 buffet, rows 1–4: *N* = 60, rows 5–7: *N* = 26, rows 8–10: *N* = 38. It was unclear how values for Age, Height, and Weight were collected and whether or not they were whole numbers. As result, we have excluded any granularity errors for those measurements.

#### Granularity errors

- Table 1, $4 buffet: Gender (male percent) (57.4, *N* = 62)
- Table 1, $4 buffet: I was hungry when I came in, mean (6.62, *N* = 62)
- Table 1, $4 buffet: I am hungry now, mean (1.88, *N* = 62)
- Table 1, $8 buffet: Gender (male percent) (47.9, *N* = 60)
- Table 1, $8 buffet: I was hungry when I came in, mean (6.64, *N* = 60)
- Table 2, $4 buffet: The middle piece of pizza I ate was very enjoyable, mean (6.64, *N* = 41)
- Table 2, $4 buffet: The last piece of pizza I ate was very satisfying, mean (6.16, *N* = 47)
- Table 2, $8 buffet: The pizza, in general, tasted really great, mean (7.44, *N* = 60)
- Table 2, $8 buffet: The first piece of pizza I ate was very satisfying, mean (7.34, *N* = 60)
- Table 2, $8 buffet: The middle piece of pizza I ate tasted really great, mean (7.97, *N* = 26)
- Table 2, $8 buffet: The middle piece of pizza I ate was very satisfying, mean (7.97, *N* = 26)
- Table 2, $8 buffet: The middle piece of pizza I ate was very enjoyable, SD (1.22, *N* = 26)
- Table 2, $8 buffet: The last piece of pizza I ate was very satisfying, mean (7.41, *N* = 38)

#### Test statistics

- Table 1, Age, *F* statistic, Reported: 0.42, Possible: 0.39–0.40
- Table 1, Age, *p* value, Reported: 0.52, Possible: 0.53–0.53
- Table 1, Number in group, *F* statistic, Reported: 1.34, Possible: 1.08–1.27
- Table 1, Number in group, *p* value, Reported: 0.25, Possible: 0.26–0.30
- Table 2, The middle piece of pizza I ate tasted really great, *F* statistic, Reported: 15.42, Possible: 13.41–14.04
- Table 2, The middle piece of pizza I ate was very satisfying, *F* statistic, Reported: 14.69, Possible: 13.41–14.04
- Table 2, The middle piece of pizza I ate was very enjoyable, *F* statistic, Reported: 12.48, Possible: 11.07–11.62

#### Visual summary

Inconsistencies are marked in red in our reproductions of Tables 1 and 2 from Article 1.


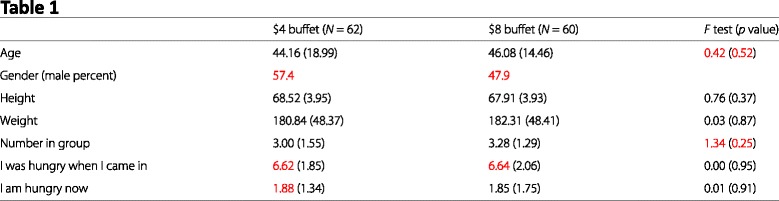



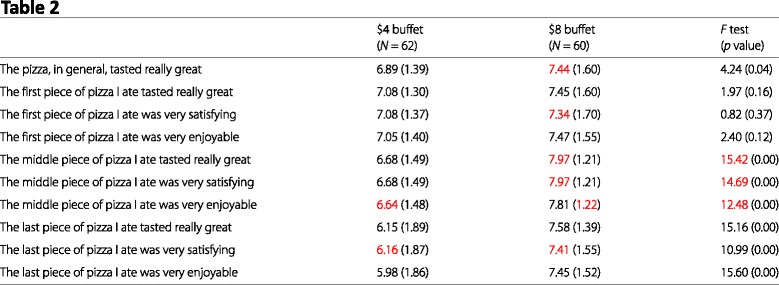


#### Miscellaneous

- Impossible degrees of freedom: “F[1,122] = 4.24; *P* = 0.04” implies the total number of diners is 124, which is more than the reported 122.
- Changing degrees of freedom throughout Table 3 analyses (can only be explained by some diners not completing the survey, which is not mentioned in the text):
  - “first piece and second piece (F[1,32] = 5.57, *P* = 0.02, *r* = 0.39”
  - “first piece and second piece...(F[1,33] = 3.77, *P* = 0.06, *r* = 0.32”
  - “first piece and second piece (F[1,35] = 0.95, *P* = 0.33, *r* = 0.17”

### Article 2: “Peak-end pizza: prices delay evaluations of quality”

Table 1 is copied verbatim from Article 1 and contains the same errors as that table. These errors are not listed again here.

#### Issues with the regression models

In the regression models in Article 2, the dependent variable (overall evaluation of all the of the slices of pizza consumed) seems to be conceptually almost indistinguishable from the predictors (the evaluation of the individual slices). It is hard to imagine any major source of variance in participants’ answers to “how much did you enjoy the pizza you ate?” apart from “how much did you enjoy each slice of pizza you ate?”; indeed, a model that included only the first slice of pizza eaten explained 97% of the variance (adjusted *R* ^2^) in the overall evaluation of the pizza. This also suggests that the predictors (i.e., the evaluations by the same person of multiple slices of pizza that they ate) cannot be regarded as independent observations; one would naturally expect the ratings by the same person of three slices of pizza, freely consumed at the same restaurant during the same visit, to be highly correlated, leading to acute problems with multicollinearity. Indeed, the authors themselves appear to have acknowledged this in the analyses that led to their Figure 1, where the degrees of freedom show that they used repeated-measures ANOVAs to determine whether the decline in ratings of the pizza from the first to the last slice was statistically significant.

#### Miscellaneous

- Impossible degrees of freedom. This test (the *F* statistic of the regression model ANOVA) for taste with the Peak-End model at $4 was reported: F(2,60) = 90.93, *p* < 0.01. This implies a sample size of 63. The sample size for this model from Table 2 is only 47, so these DFs should have been reported as (2,44).
- Incorrect degrees of freedom. This test (the *F* statistic of the regression model ANOVA) for pizza satisfaction with the Peak-End model at $4 was reported: F(2,42) = 37.25, *p* < 0.01. This implies a sample size of 45. The sample size for this model should be the same as the pizza taste model, which was 47, so these DFs should have been reported as (2,44).

### Article 3: “Eating heavily: men eat more in the company of women”

As with Article 1, Age, Weight, and Height were ignored for granularity testing. It also wasn’t clear what values “salad consumed” and “pizza slices consumed” could take, as a result those are also excluded from granularity testing. The first column of Table 3 is the same as the second column in Table 2. Those errors will not be listed twice, but will be shown in the visual summary.

#### Granularity errors

- Table 2, Males eating with females, I felt rushed, mean (1.46, *N* = 40)
- Table 2, Males eating with females, I am physically uncomfortable, mean (2.11, *N* = 40)
- Table 2, Males eating with males, I overate, mean (2.76, *N* = 20)
- Table 2, Males eating with males, I am physically uncomfortable, mean (2.27, *N* = 20)
- Table 2, Females eating with males, I overate, mean (2.73, *N* = 35)
- Table 2, Females eating with males, How many calories..., mean (463.61, *N* = 35)
- Table 2, Females eating with females, I felt rushed, mean (1.18, *N* = 10)
- Table 2, Females eating with females, I felt rushed, SD (0.40, *N* = 10)
- Table 2, Females eating with females, How many calories..., mean (111.71, *N* = 10)
- Table 2, Females eating with females, I am physically uncomfortable, mean (1.91, *N* = 10)
- Table 3, Only one male in mixed-sex groups, I overate, mean (2.92, *N* = 21)
- Table 3, Only one male in mixed-sex groups, I felt rushed, mean (1.65, *N* = 21)
- Table 3, Only one male in mixed-sex groups, I am physically uncomfortable, mean (2.32, *N* = 21)
- Table 3, More than one male in mixed-sex groups, I felt rushed, SD (1.23, *N* = 19)
- Table 3, More than one male in mixed-sex groups, I am physically uncomfortable, SD (1.24, *N* = 19)

#### Test statistics

- Table 1, Age, Males, *t* statistic, Reported: 0.42, Possible: 0.22–0.22 (Means were assumed to be 44.00 and 43.00)
- Table 1, Height, Males, *t* statistic, Reported: 1.59, Possible: 1.48–1.49
- Table 1, Weight, Males, *t* statistic, Reported: 2.87, Possible: 2.76–2.76
- Table 1, BMI, Males, *t* statistic, Reported: 2.52, Possible: 2.43–2.43
- Table 1, Age, Females, *t* statistic, Reported: 0.64, Possible: 0.60–0.60
- Table 1, Height, Females, *t* statistic, Reported: 0.37, Possible: 0.38–0.38
- Table 1, Weight, Females, *t* statistic, Reported: 2.38, Possible: 2.70–2.70
- Table 1, BMI, Females, *t* statistic, Reported: 2.96, Possible: 3.36–3.39
- Table 2, Salad consumed, Effect of gender, *F* statistic, Reported: 3.84, Possible: 4.64–4.81
- Table 2, Pizza slices consumed, Effect of gender, *F* statistic, Reported: 14.58, Possible: 12.41–13.07
- Table 2, How many calories..., Effect of gender, *F* statistic, Reported: 5.01, Possible: 6.94–6.94
- Table 2, I am physically uncomfortable, Effect of gender, *F* statistic, Reported: 0.15, Possible: 0.11–0.14
- Table 2, Salad consumed, Effect of group type, *F* statistic, Reported: 1.36, Possible: 1.64–1.73
- Table 2, Pizza slices consumed, Effect of group type, *F* statistic, Reported: 9.26, Possible: 7.83–8.32
- Table 2, How many calories..., Effect of group type, *F* statistic, Reported: 10.39, Possible: 14.38–14.38
- Table 2, Salad consumed, Effect of gender ×group, *F* statistic, Reported: 4.83, Possible: 5.90–6.10
- Table 2, Pizza slices consumed, Effect of gender ×group, *F* statistic, Reported: 4.22, Possible: 3.52–3.83
- Table 2, I overate, Effect of gender ×group, *F* statistic, Reported: 4.15, Possible: 3.89–4.10
- Table 2, How many calories..., Effect of gender ×group, *F* statistic, Reported: 4.05, Possible: 5.61–5.62
- Table 2, I am physically uncomfortable, Effect of gender ×group, *F* statistic, Reported: 0.39, Possible: 0.31–0.38
- Table 3, How many calories..., *F* statistic, Reported: 0.15, Possible: 2.26–2.26
- Table 3, I am physically uncomfortable, *F* statistic, Reported: 0.72, Possible: 0.28–0.32

#### Visual summary

Inconsistencies are marked in red in our reproductions of Tables 1, 2, and 3 from Article 3.


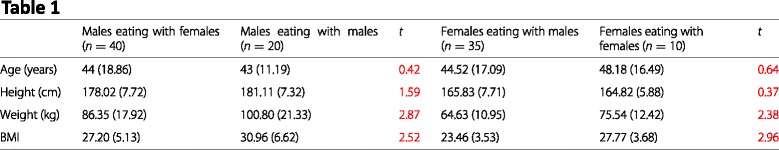



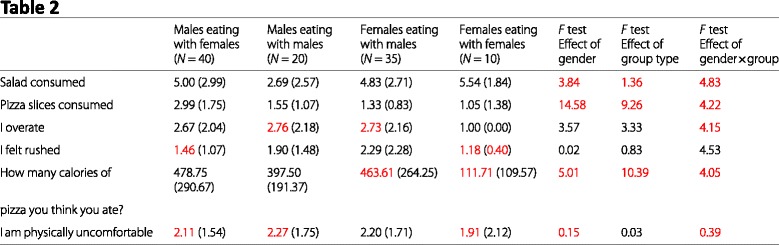



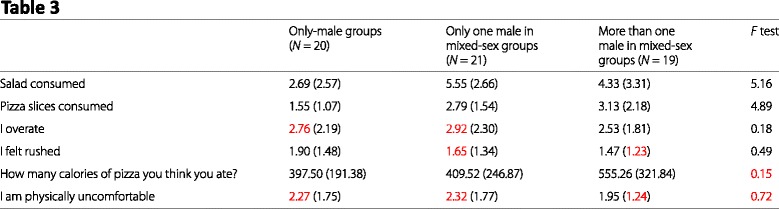


#### Miscellaneous

- Table 1, Males eating with females, Weight kg to pounds conversion, Reported: 191.89, Possible: 190.36–190.38
- Table 1, Males eating with males, Height cm to inches conversion, Reported: 71.28, Possible: 71.30–71.31
- Table 1, Males eating with males, Weight kg to pounds conversion, Reported: 224.00, Possible: 222.21–222.24
- Table 1, Females eating with males, Weight kg to pounds conversion, Reported: 143.62, Possible: 142.47–142.50
- Table 1, Females eating with females, Height cm to inches conversion, Reported: 64.83, Possible: 64.89–64.89
- Table 1, Females eating with females, Weight kg to pounds conversion, Reported: 167.28, Possible: 166.53–166.55
- Impossible degrees of freedom. These DFs are provided for a 2x2 ANOVA: “(1,109)”. This implies a sample size of 109+(2)(2)=113 while the total number of diners in this article is 105.
- Changing degrees of freedom. For the same 2x2 ANOVA that listed the DFs “(1,109)”, the DFs “(1,98)”, “(1,115)”, and “(1,112)” are also used. None of these DFs match the total number of 105 diners.
- The SD for I overate, Males eating with males, changes between Tables 8 and 9 (2.18 versus 2.19).

### Article 4: “Low prices and high regret: how pricing influences regret at all-you-can-eat buffets”

As noted in the text of our article, there are inconsistencies in the sample sizes of some of the columns in Tables 11 and 12. Where a sample size was identical between these two tables, any granularity errors in that column are listed only once here, although both cases are highlighted in the visual summary. Where the sample size changed, granularity tests were performed on the entire column in both tables. As with Articles 1 and 3, Age, Weight, and Height were ignored for granularity testing.

#### Granularity errors

- Table 2, I ate more pizza than I should have, $4, One piece, mean (2.63, *N* = 18)
- Table 2, I am physically uncomfortable, $4, One piece, SD (1.88, *N* = 18)
- Table 2, I ate more pizza than I should have, $4, Two pieces, mean (4.82, *N* = 18)
- Table 2, I feel guilty about how much I ate, $4, Two pieces, SD (2.47, *N* = 18)
- Table 2, I am physically uncomfortable, $4, Two pieces, SD (2.12, *N* = 18)
- Table 2, I feel guilty about how much I ate, $4, Three pieces, SD (1.49, *N* = 7)
- Table 2, I overate, $4, Three pieces, SD (1.79, *N* = 7)
- Table 2, I ate more than I should have, $4, Three pieces, SD (2.22, *N* = 7)
- Table 2, I feel guilty about how much I ate, $8, One piece, mean (2.26, *N* = 17)
- Table 2, I am physically uncomfortable, $8, One piece, mean (1.97, *N* = 17)
- Table 2, I overate, $8, One piece, mean (1.67, *N* = 17)
- Table 2, I ate more than I should have, $8, One piece, SD (1.45, *N* = 17)
- Table 2, I am physically uncomfortable, $8, Two pieces, mean (1.45, *N* = 19)
- Table 2, I overate, $8, Two pieces, mean (1.67, *N* = 19)
- Table 2, I ate more than I should have, $8, Two pieces, mean (2.14, *N* = 19)
- Table 2, I am physically uncomfortable, $8, Three pieces, mean (2.25, *N* = 10)
- Table 2, I overate, $8, Three pieces, SD (2.74, *N* = 10)
- Table 2, I ate more than I should have, $8, Three pieces, mean (3.92, *N* = 10)
- Table 3, I ate more pizza than I should have, $8, One piece, mean (1.76, *N* = 19)
- Table 3, I am physically uncomfortable, $8, One piece, mean (1.955, *N* = 19)
- Table 3, I overate, $8, One piece, mean (1.67, *N* = 19)
- Table 3, I ate more pizza than I should have, $8, Two pieces, mean (3.53, *N* = 21)
- Table 3, I feel guilty about how much I ate, $8, Two pieces, mean (1.68, *N* = 21)
- Table 3, I am physically uncomfortable, $8, Two pieces, mean (1.28, *N* = 21)
- Table 3, I overate, $8, Two pieces, mean (1.53, *N* = 21)
- Table 3, I ate more pizza than I should have, $8, Three pieces, mean (4.40, *N* = 12)
- Table 3, I feel guilty about how much I ate, $8, Three pieces, mean (2.90, *N* = 12)
- Table 3, I am physically uncomfortable, $8, Three pieces, mean (2.10, *N* = 12)
- Table 3, I overate, $8, Three pieces, SD (2.95, *N* = 12)

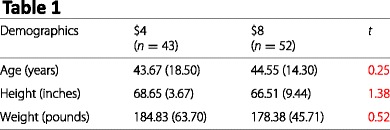



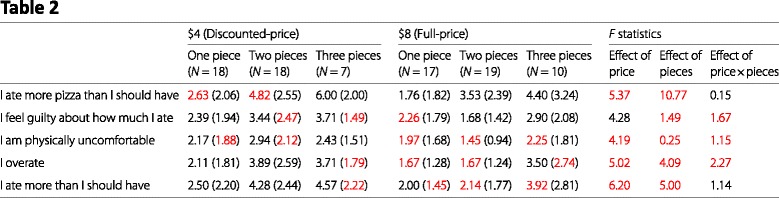



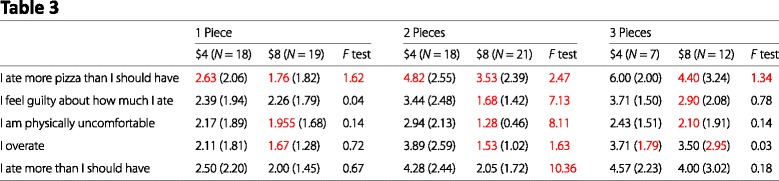


#### Test statistics

- Table 1, Height (inches), *t* statistic, Reported: 1.38, Possible: 1.39–1.41
- Table 1, Weight (pounds), *t* statistic, Reported: 0.52, Possible: 0.57–0.57
- Table 2, I ate more pizza than I should have, Effect of Price, *F* statistic, Reported: 5.37, Possible: 5.41–5.63
- Table 2, I am physically uncomfortable, Effect of Price, *F* statistic, Reported: 4.19, Possible: 2.49–2.69
- Table 2, I overate, Effect of Price, *F* statistic, Reported: 5.02, Possible: 4.61–4.86
- Table 2, I ate more than I should have, Effect of Price, *F* statistic, Reported: 6.20, Possible: 5.04–5.28
- Table 2, I ate more pizza than I should have, Effect of Pieces, *F* statistic, Reported: 10.77, Possible: 10.80–2.05
- Table 2, I feel guilty about how much I ate, Effect of Pieces, *F* statistic, Reported: 1.49, Possible: 1.77–1.87
- Table 2, I am physically uncomfortable, Effect of Pieces, *F* statistic, Reported: 0.25, Possible: 0.15–0.18
- Table 2, I overate, Effect of Pieces, *F* statistic, Reported: 4.09, Possible: 4.99–5.16
- Table 2, I ate more than I should have, Effect of Pieces, *F* statistic, Reported: 5.00, Possible: 5.61–5.78
- Table 2, I feel guilty about how much I ate, Effect of Price ×pieces, *F* statistic, Reported: 1.67, Possible: 1.13–1.20
- Table 2, I am physically uncomfortable, Effect of Price ×pieces, *F* statistic, Reported: 1.15, Possible: 1.21–1.30
- Table 2, I overate, Effect of Price ×pieces, *F* statistic, Reported: 2.27, Possible: 2.03–2.14
- Table 3, I ate more pizza than I should have, One piece, *F* statistic, Reported: 1.62, Possible: 1.81–1.91
- Table 3, I ate more pizza than I should have, Two pieces, *F* statistic, Reported: 2.47, Possible: 2.60–2.71
- Table 3, I ate more pizza than I should have, Three pieces, *F* statistic, Reported: 1.34, Possible: 1.36–1.40
- Table 3, I feel guilty about how much I ate, Two pieces, *F* statistic, Reported: 7.13, Possible: 7.54–7.79
- Table 3, I am physically uncomfortable, Two pieces, *F* statistic, Reported: 8.11, Possible: 11.93–12.36
- Table 3, I overate, Two pieces, *F* statistic, Reported: 1.63, Possible: 14.62–15.01
- Table 3, I ate more than I should have, Two pieces, *F* statistic, Reported: 10.36, Possible: 10.97–11.27

#### Visual summary

Inconsistencies are marked in red in our reproductions of Tables 1, 2, and 3 from Article 4.

#### Miscellaneous

- The following entries change between Tables 2 and 3:
  - One piece, $8, Sample size
  - Two pieces, $8, Sample size
  - Three pieces, $8, Sample size
  - I feel guilty about how much I ate, Two pieces, $4, SD
  - I feel guilty about how much I ate, Three pieces, $4, SD
  - I am physically uncomfortable, One piece $4, SD
  - I am physically uncomfortable, Two pieces $4, SD
  - I ate more than I should have, Three pieces, $4, SD
  - I am physically uncomfortable, One piece, $8, mean
  - I am physically uncomfortable, Two pieces, $8, mean
  - I am physically uncomfortable, Two pieces, $8, SD
  - I am physically uncomfortable, Three pieces, $8, mean
  - I am physically uncomfortable, Three pieces, $8, SD
  - I overate, Two pieces, $8, mean
  - I overate, Two pieces, $8, SD
  - I overate, Three pieces, $8, SD
  - I ate more than I should have, Two pieces, $8, mean
  - I ate more than I should have, Two pieces, $8, SD
  - I ate more than I should have, Three pieces, $8, mean
  - I ate more than I should have, Three pieces, $8, SD
- The sample sizes in Table 2 do not add up to 95
- Incorrect degrees of freedom: The text describes an apparent 3 ×1 ANOVA with the DFs “(2, 84)”, implying a total of 84+3=87 diners when there are 95 diners in total
- Incorrect degrees of freedom: The text describes an apparent 2 ×1 ANOVA with the DFs “(1, 84)”, implying a total of 84+2=86 diners when there are 95 diners in total
- Table 1, Height, $8, SD seems excessively large (the SD of human height is typically around 4 inches; see also Table 1 of Article 1)
- Table 1, Weight, $4, SD is large and inconsistent with the SD in the $8 condition, as well as with the SDs in Table 1 of Article 1.

## Abbreviations

DF: Degree(s) of freedom
SD: Standard deviation
